# Daily oral consumption of hydrolyzed type 1 collagen is chondroprotective and anti-inflammatory in murine posttraumatic osteoarthritis

**DOI:** 10.1371/journal.pone.0174705

**Published:** 2017-04-06

**Authors:** Qurratul-Ain Dar, Eric M. Schott, Sarah E. Catheline, Robert D. Maynard, Zhaoyang Liu, Fadia Kamal, Christopher W. Farnsworth, John P. Ketz, Robert A. Mooney, Matthew J. Hilton, Jennifer H. Jonason, Janne Prawitt, Michael J. Zuscik

**Affiliations:** 1 Center for Musculoskeletal Research, University of Rochester Medical Center, Rochester, New York, United States of America; 2 Department of Biology, University of Rochester, Rochester, New York, United States of America; 3 Department of Pathology and Laboratory Medicine, University of Rochester Medical Center, Rochester, New York, United States of America; 4 Department of Orthopaedics & Rehabilitation, University of Rochester Medical Center, Rochester, New York, United States of America; 5 Orthopaedic Cellular, Developmental & Genome Laboratories, Duke University, Durham, North Carolina, United States of America; 6 Rousselot BVBA, Gent, Belgium; Ohio State University, UNITED STATES

## Abstract

Osteoarthritis (OA) is a degenerative joint disease for which there are no disease modifying therapies. Thus, strategies that offer chondroprotective or regenerative capability represent a critical unmet need. Recently, oral consumption of a hydrolyzed type 1 collagen (hCol1) preparation has been reported to reduce pain in human OA and support a positive influence on chondrocyte function. To evaluate the tissue and cellular basis for these effects, we examined the impact of orally administered hCol1 in a model of posttraumatic OA (PTOA). In addition to standard chow, male C57BL/6J mice were provided a daily oral dietary supplement of hCol1 and a meniscal-ligamentous injury was induced on the right knee. At various time points post-injury, hydroxyproline (hProline) assays were performed on blood samples to confirm hCol1 delivery, and joints were harvested for tissue and molecular analyses were performed, including histomorphometry, OARSI and synovial scoring, immunohistochemistry and mRNA expression studies. Confirming ingestion of the supplements, serum hProline levels were elevated in experimental mice administered hCol1. In the hCol1 supplemented mice, chondroprotective effects were observed in injured knee joints, with dose-dependent increases in cartilage area, chondrocyte number and proteoglycan matrix at 3 and 12 weeks post-injury. Preservation of cartilage and increased chondrocyte numbers correlated with reductions in MMP13 protein levels and apoptosis, respectively. Supplemented mice also displayed reduced synovial hyperplasia that paralleled a reduction in *Tnf* mRNA, suggesting an anti-inflammatory effect. These findings establish that in the context of murine knee PTOA, daily oral consumption of hCol1 is chondroprotective, anti-apoptotic in articular chondrocytes, and anti-inflammatory. While the underlying mechanism driving these effects is yet to be determined, these findings provide the first tissue and cellular level information explaining the already published evidence of symptom relief supported by hCol1 in human knee OA. These results suggest that oral consumption of hCol1 is disease modifying in the context of PTOA.

## Introduction

Osteoarthritis (OA) is one of the most prevalent diseases in the world, with recent estimates projecting that >250 million people are afflicted globally [1]. In the US, OA afflicts 35 million people [2], with diarthrodial and spinal OA being the most prevalent disease, surpassing the next top four medical disorders combined (heart, pulmonary, mental health and diabetic conditions) [3]. In a recent analysis, global medical costs for lower extremity OA exceed $350 billion/year, with the reduced quality of life and physical function of OA patients exerting an additional hidden economic impact that surpasses $50 billion/year [4]. Despite these statistics, there are no disease modifying therapies available, with management of OA patients consisting only of symptom palliation [5, 6] via nonsteroidal anti-inflammatory drugs, intraarticular injection of corticosteroids or hyaluronic acid, narcotic-based analgesia including opioids, and joint arthroplasty. Thus, the development of therapeutic strategies that offer protective and/or regenerative capability is a critical unmet need and central pursuit in the OA field.

OA is a joint disease of multifactorial etiology characterized by degeneration and loss of articular cartilage and meniscus, subchondral bone sclerosis, osteophyte formation, and synovial hyperplasia [7–9]. Etiologic complexity and ‘whole organ’ involvement of multiple tissues within the OA joint during the degenerative process represent significant challenges in the development of disease modifying therapeutic strategies. Over the past two decades, more than a dozen human clinical trials have been performed to test candidate disease modifying OA drugs (DMOADs), none of which have emerged to be accepted as a bona fide therapeutic agent [10, 11]. This list includes orally administered nutraceutical agents comprised of cartilage matrix components that are widely marketed as joint protective. In the case of chondroitin sulfate and glucosamine, the two most widely available and top selling formulations, mixed clinical trial results leave open the question of their efficacy [12].

An agent with a reported positive influence on chondrocyte function and potential disease modifying capability in OA is hydrolyzed type 1 collagen (referred to in this report as hCol1). hCol1 is a mixture of type I collagen peptides of different molecular weights that are generated via enzymatic digestion of type I collagen extracted from animal connective tissues. The peptide mixture, which contains an abundance of hydroxyproline, proline and glycine, is absorbed dose-dependently following bolus oral delivery [13], with a series of di- and tri-peptides peaking in the circulation within one hour after consumption in humans [14]. hCol1 is considered safe as an oral supplement [15], and when consumed daily, beneficial effects have been observed in bone [16] and skin [17, 18]. Recent data suggesting that hCol1 has chondrogenic effects in vitro [19] is consistent with the growing consensus that collagen derivatives may be chondroprotective in OA [20] and aligns with recent clinical evidence of its symptom-relieving effects in human subjects suffering from knee joint pain [21]. Overall, based on this body of data, in this study we posed the hypothesis that daily oral supplementation with hCol1 will have protective effects in joints undergoing the OA degenerative process. To address this hypothesis and characterize the joint protective effect(s) of hCol1, we conducted a preclinical study examining the impact of this agent, at the tissue and cellular level, in an established mouse model of posttraumatic knee OA (PTOA).

## Materials and methods

### Animals

**Fig 1:**
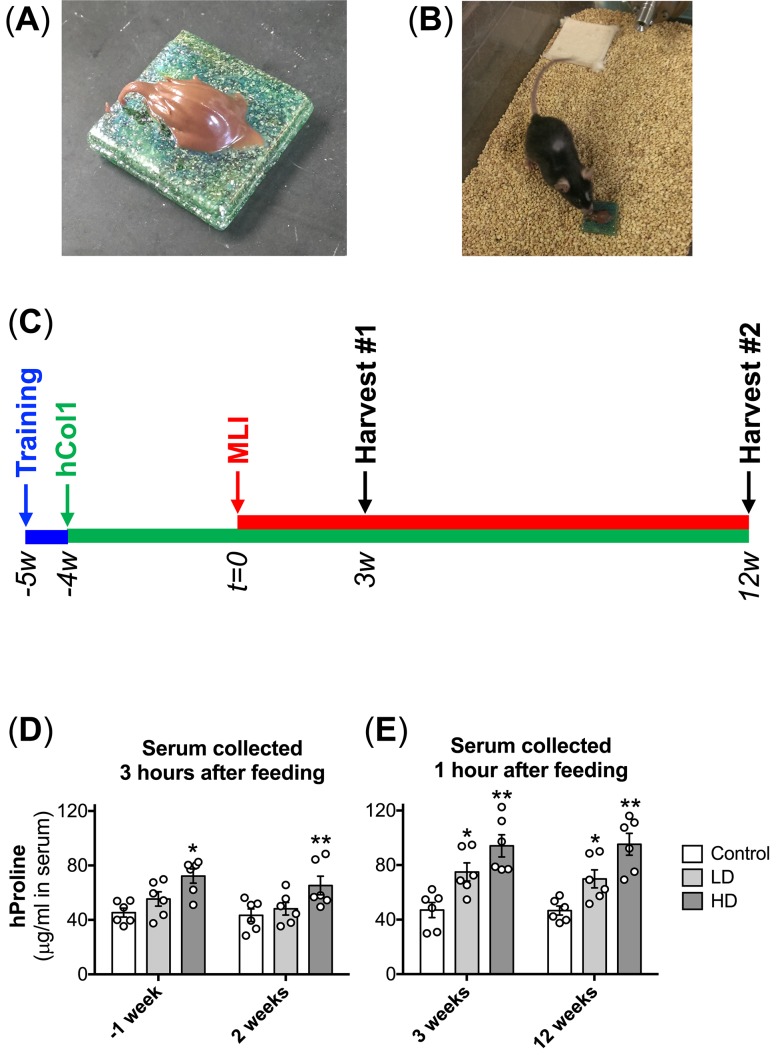
Effective bolus delivery of hCol1 and experimental timeline. Hazelnut cream was used as a vehicle to deliver daily bolus doses of hCol1 to mice such that a delivery of a 150mg mixture provided a daily bolus dose of either 3.8mg (LD) or 38mg (HD) hCol1 (Control = hazelnut cream alone). Experimental mixtures were placed on autoclavable ceramic tiles (A) and presented to individually house mice (B) at the same time daily. After 5–7 days of training with vehicle alone, mice consumed the full amount presented within 2 minutes. Panel (C) depicts the experimental timeline. Mice were presented hazelnut cream daily in the bolus feeding regimen for a 1 week training period (blue line), and then Control, LD and HD daily supplements were initiated and continued for the remainder of the experiment (green line). After 4 weeks of supplementation, MLI (right knee) and Sham (left knee) surgery was performed (t = 0), followed by tissue harvests at 3 weeks and 12 weeks post-surgery. (D) To confirm successful delivery of hCol1, serum hProline levels were quantified via ELISA. Serum samples collected 1 week before (-1) and 2 weeks after surgery were harvested 3 hours after the mice consumed supplements (left graph). Serum samples collected 3 and 12 weeks after surgery were harvested 1 hour after consumption of the supplements (right graph). Symbols (○) represent the hProline level in the serum of individual mice. Bars represent the average hProline level for each experimental group (± SEM, N = 6). Significant differences between groups were identified via two-way ANOVA with a Tukey’s multiple comparisons post-test (*p<0.05, **p<0.01 compared to Control).

All handling of mice and in vivo experimental procedures performed in studies reported here were reviewed and approved by the Institutional Animal Care and Use Committee (IACUC) at the University of Rochester (protocol number UCAR-2005-226R). Male C57BL/6J mice were purchased from Jackson Laboratories and were housed individually in micro-isolator cages on a 12 hour light/dark cycle. Male mice were used in this study due to a faster and more temporally predictable progression of degeneration that has been documented in models of PTOA [22]. Mice had ad libitum access to standard chow and fresh water, and were supplemented with hCol1 of bovine origin and a mean molecular weight of 2000 Da (Peptan^®^ B2000, Rousselot) using a method previously described to deliver daily doses of estradiol [23]. Briefly, hCol1 was incorporated into hazelnut cream such that 150mg of the mixture would deliver either low dose (LD, 3.8mg) or high dose (HD, 38mg) hCol1 when completely consumed. The HD is the body weight adjusted mouse equivalent to the 7.4g/day recommended human dose. At the beginning of the experimental time line (Fig 1C), 12 week old mice were presented with an autoclavable ceramic tile loaded with a 150mg aliquot of hazelnut cream vehicle, LD hCol1 or HD hCol1 (Fig 1A). These experimental supplements were provided daily at the same time (in the morning), and once trained, the mice consumed the entire provided amount within 2 minutes (Fig 1B). Modeling the daily consumption regimen suggested for lifelong joint health in humans, mice were fed the supplements daily during the entire experimental protocol until collection of experimental endpoints (Fig 1D).

To initiate PTOA, we employed a method developed and routinely used by our group known as meniscal-ligamentous injury (MLI) [24–27]. Briefly, mice in the experimental protocol (at 17 weeks of age) were anesthetized via intraperitoneal injection of 60mg/kg ketamine and 4mg/kg xylazine and after creating a 3mm incision over the anteromedial aspect of the right knee joint, the medial collateral ligament of the right knee was transected and a segment of the anterior horn of the medial meniscus was excised. This injury leads to detectable PTOA joint changes by 4 weeks post-injury and progresses over 4 months [24], similar to that seen in the DMM model of posttraumatic OA [28]. The contralateral limb provided a sham control, with the surgery consisting of only the incision (no joint structures were manipulated). Mice were provided buprenorphine analgesia (0.5mg/kg) at the time of surgery and every 12 hours for 3 days.

### Tissue fixation and histology preparation

A previously established systematic approach to preparation, sectioning and visualizing mouse knee joint articular cartilage was employed for all tissue-based assays [24]. At the time of harvest (3 or 12 weeks post-MLI), mice were sacrificed using an AMVA-approved method and the knee joints were dissected with the femur and tibia intact to maintain joint structure. Tissues were fixed in 4% paraformaldehyde at 4°C for 72 hours, decalcified in 5% formic acid for 10 days, processed using a microwave processor, and embedded in paraffin. Tissue blocks were then serially sectioned in the midsagittal plane through the medial compartment of the joint. A series of 5μm thick sections were cut at three distinct levels within the medial compartment, mounted on positively-charged glass slides, baked at 60°C overnight, de-paraffinized in xylene, and rehydrated in decreasing concentrations of ethanol. To support study of tissue structure via histomorphometry and to accommodate OARSI and Synovial scoring methods, mounted sections were stained with either Toluidine Blue/Fast Green (0.04%/0.02%), Alcian Blue Hematoxylin/Orange G or Safranin O/Fast Green (1%/0.02%) using optimized protocols established by the Histology, Biochemistry and Molecular Imaging Core in the Center for Musculoskeletal Research at the University of Rochester. Unstained sections were used for the various molecular and cellular analyses described below.

### Cartilage histomorphometry

Two different approaches were used to quantify cartilage content. An automated method to quantify tissue architecture in murine allograft healing [29] was modified and utilized to determine total cartilage area (uncalcified plus calcified) using Toluidine Blue/Fast Green-stained sections. After scanning to high resolution digital files using an Olympus VS120 Virtual Slide Microscope/Slide Scanner system, histologic images were analyzed using a software-based application that was developed to automatically distinguish between bone and cartilage based on the contrasting stain differential between these tissues (Toluidine Blue-stained cartilage and Fast Green-stained subchondral bone). Experimental image files were serially analyzed to quantify total cartilage area on the tibial plateau and femoral condyle, with the application returning information about each area separately. To compliment this, we also performed manual histomorphometry using the OsteoMetrics System as we have previously published [24, 26]. Briefly, Safranin O/Fast Green stained sections were individually viewed using an Olympus BH2 light microscope interfaced with the OsteoMetrics System via a digital camera. OsteoMeasureXP software facilitated quantification of uncalcified cartilage, calcified cartilage and chondrocyte populations. Articular cartilage areas examined on the tibial plateau and femoral condyle were defined to be between the anterior and posterior horns of the meniscus, using a region of interest of defined size for all sections that were analyzed.

### OARSI and synovial scoring

Regarding cartilage, semi-quantitative histopathologic grading was performed using a scoring system that has been established by the OARSI histopathology initiative as the standard method for grading of mouse cartilage degeneration [30]. Based on this system, cartilage grading was carried out using Alcian Blue Hematoxylin/Orange G-stained joint sections using the following scale: 0 = normal cartilage, 0.5 = loss of proteoglycan stain without cartilage damage, 1 = mild superficial fibrillation, 2 = fibrillation and/or clefting extending below the superficial zone, 3 = mild (<25%) loss of cartilage, 4 = moderate (25–50%) loss of cartilage, 5 = severe (50–75%) loss of non-calcified cartilage, and 6 = eburnation with >75% loss of cartilage. Synovial scores were also obtained from each joint section, with the score reporting on the degree of synovial hyperplasia (i.e. thickness and cellularity of the synovial membrane) as we have previously performed [31]. Briefly, a subjective scoring system of 0 to 2 was employed: 0 = a synovial lining that is several (2–3) cell layers thick or <10μm thick (normal), 1 = synovial thickening with a lining cell layer between 5 and 10 cells thick or between 10μm and 20μm thick, and 2 = severe thickening of the synovial lining >10 cells and/or >20μm thick. OARSI and synovial scoring was performed by four blinded observers (QAD, EMS, RAM and MJZ) and observer agreement for each score was evaluated in pairs via calculation of a weighted kappa coefficient, using Fleiss-Cohen weights, as we have described [24]. The average pairwise coefficient was 0.90, indicative of strong agreement between the observers. The four scores (OARSI and synovium) for each section were averaged and the data from each group of mice were combined.

### Molecular analysis of tissues

Mouse knee joint sections evaluated by immunohistochemistry were treated as follows: Endogenous peroxidases were quenched with BLOXALL (Vector) for 10 minutes followed by treatment with 3% hydrogen peroxide for 20 min, and a 1:20 dilution of normal goat serum for 30 min. Slides were incubated overnight at 4°C with a rabbit anti-mouse TNF polyclonal (1:200; Abcam #ab6671) or a rabbit anti-human Ki-67 monoclonal antibody (1:200; Abcam #ab66155). For TNF detection, slides were then rinsed with phosphate-buffered saline containing 0.5% Tween 20, and incubated for 30 minutes at room temperature with a biotinylated goat anti-rabbit IgG (1:200; Vector). Antibody binding to TNF was detected following application of ABC reagent from Vectastain Elite ABC Kit (Vector) for 30 minutes, with a 5 minute application of ImmPACT DAB Peroxidase (HRP) Substrate (Vector) for 5 minutes. Nuclei were counterstained for 20 seconds with Mayer’s Hematoxylin (Biocare Medical). For detection of Ki-67, after overnight incubation with primary antibody, slides were rinsed with phosphate-buffered saline and incubated for 30 minutes at room temperature with Alexa Fluor 488-conjugated goat anti-rabbit IgG (1:250; Invitrogen #ab150077). Apoptotic chondrocytes were identified using the TUNEL In Situ Cell Death Detection Kit (Roche, #11684795910) as instructed by the manufacturer. In the case of Ki-67 and TUNEL, nuclei were counterstained with DAPI and experimental fluorescence was imaged using a Zeiss Axioskop 40 BF/DF/Fluorescence Microscope with a SPOT RT3 Color/Slider Camera.

### qRTPCR analysis of synovial TNF expression

At sacrifice, synovial capsules were harvested from experimental joints with the aid of surgical loops. Recovered synovial tissue was stored at -80°C until extraction of total mRNA. mRNA was isolated from individual capsules using the Qiagen RNeasy Fibrous Tissue mini kit (Qiagen) using the manufacturer’s instructions. One μg of total RNA was used to synthesize cDNA using the iScript cDNA Synthesis Kit (BioRad). The abundance of mouse *β-actin*, *Tnf*, *Il1β*, *Mmp13* and *Prg4* was then assessed by qRTPCR using SYBR Green Real Time PCR Master Mix (Qiagen). Reactions were carried out using a Rotor Gene 6000 PCR machine. Forward and reverse primer sequences for each of these gene transcripts are reported in Table 1.

**Table 1:** qRTPCR primer sequences.

Transcript	Forward Primer	Reverse Primer
*Tnf*	5’-CTCTTCTGTCTACTGAACTTCGGG-3’	5’-GAGAAGATGATCTGAGTGTGAGGG-3’
*Il1β*	5’-CACAGCAGCACATCAACAAG-3’	5’-GTGCTCATGTCCTCATCCTG-3’
*Mmp13*	5’-AAGATGTGGAGTGCCTGATG-3’	5’-AAGGCCTTCTCCACTTCAGA-3’
*Prg4*	5’-AGTGCTGTCCTGATTTCAAGAG-3’	5’-GGTGATTTGGGTGAGCGTTTGGTA-3’
*β-actin*	5’-TGTTACCAACTGGGACGACA-3’	5’-CTGGGTCATCTTTTCACGGT-3’

### Statistical analyses

For all histomorphometry-based analysis of cartilage architecture, including cartilage area and chondrocyte population studies, one-way ANOVA with a Tukey’s multiple comparisons post-test was performed. After confirming blinded observer agreement via calculation of weighted kappa coefficients, OARSI and synovial scoring studies were analyzed using a Kruskal-Wallis Test with a Dunn’s multiple comparisons post-test. These analyses and all graphing of the data were carried out using Graphpad Prism software. Differences between groups were considered significant when a *p*-value <0.05 was achieved.

## Results

### Strategy for bolus delivery of hCol1

Daily bolus delivery of hCol1 over the course of the 16 week experimental time line (Fig 1C) was used to investigate joint protective effects that we hypothesize are supported by long term daily consumption of hCol1 to support joint health. To support the daily bolus delivery strategy, hCol1 was incorporated into hazelnut cream (Nutella^®^) such that a 150mg amount of the experimental mixtures would support delivery of 3.8mg (LD) or 38mg (HD) of hCol1. Once prepared, 150mg aliquots of Control (hazelnut cream vehicle alone), LD and HD hCol1 were deposited onto sterile ceramic tiles (Fig 1A) and presented to each individually housed mouse at the same time in the morning daily (Fig 1B). Mice typically consumed the entire supplement within 2 minutes, supporting bolus delivery. To confirm successful absorption of the hCol1 supplements into the circulation, blood samples were collected from mice 1 week before and 2, 3, and 12 weeks after surgery (sham or MLI, Fig 1C). For the first two time points, the blood draw was collected 3 hours after consumption of the supplements, while the second two time points involved a blood draw 1 hour after consumption. As expected, serum levels of hProline were increased following delivery of the hCol1 supplements. When collected 3 hours after bolus delivery, only the HD hCol1 group displayed a significant increase in serum hProline levels (Fig 1D). Comparatively, when blood draws were collected 45 minutes after bolus delivery, both LD and HD groups displayed significant increases in serum hProline levels, with apparent dose dependency (Fig 1E). These results confirm that the bolus delivery regimen was effective, with increased serum levels of hProline establishing increased absorption of hCol1 following both LD and HD hCol1 supplementation.

### Impact of hCol1 supplements on cartilage structure

**Fig 2:**
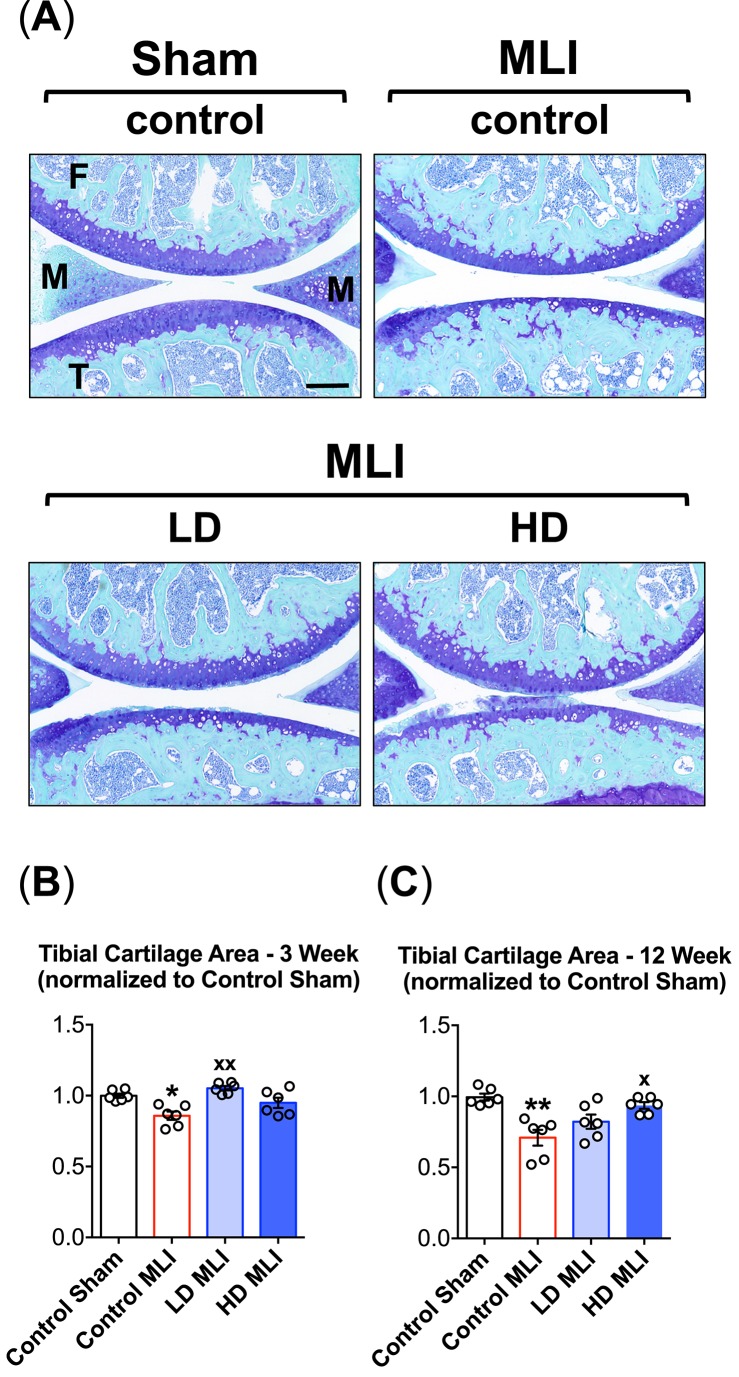
Cartilage loss following MLI in hCol1-fed mice is reduced. Panel (A) presents an array of representative 40x Toluidine Blue/Fast Green stained sagittal sections from the medial compartment of sham and MLI joints 12 weeks post-injury under various treatment conditions (control = vehicle, LD = 3.8mg hCol1/day, HD = 38mg hCol1/day). Joint structures are labeled (F = femur, M = meniscus, T = tibia) and the black scale bar depicts 100μm. Total tibial cartilage area was determined in these representative sections as well as a series of similarly stained serial sections from all experimental joints at both 3 weeks (B) and 12 weeks (C) post-MLI using an automated approach (Visiopharm System). Symbols (○) represent the average tibial cartilage area of 3 sections/joint. Bars represent the average tibial cartilage area for each experimental group (± SEM, N = 6). Significant differences between experimental groups were identified via one-way ANOVA with a Tukey’s multiple comparisons post-test (*p<0.05, **p<0.01 compared to Control Sham; ^**x**^p<0.05, ^**xx**^p<0.01 compared to Control MLI).

Initial analysis of the knee joint articular cartilage involved automated histomorphometry to determine total cartilage area in sagittal sections stained with Toluidine Blue and Fast Green. Stained sections were digitized to images and an ‘Articular Cartilage Application’ was developed on the Visiopharm Platform to streamline the determination of total cartilage area on the femoral condyle and tibial plateau. As we have seen previously in the MLI model of PTOA [24–27], the main cartilage changes played out on the tibial plateau, where there was significant total cartilage loss detectable at both 3 weeks (Fig 2B) and 12 weeks (Fig 2A and 2C) post injury. No changes in femoral condyle articular cartilage area were detected at either 3 or 12 weeks after injury or in any of the experimental groups (data not shown). Suggesting possible chondroprotective effects of hCol1 supplementation, total tibial cartilage area loss 3 weeks after MLI was prevented in the LD hCol1 group, with the HD group trending toward improvement but missing significance (Fig 2B). At the 12 weeks post-MLI time point, hCol1 was dose-dependently protective, with total tibial cartilage area loss mitigated significantly in the HD group, and the LD group trending toward significance (Fig 2C). It should be noted that there were no discernable effects of hCol1 supplementation on baseline (Sham-operated) articular cartilage area (data not shown). Overall, these results provide the first indication that dietary supplementation with hCol1 could be protective in the development and progression of PTOA.

**Fig 3:**
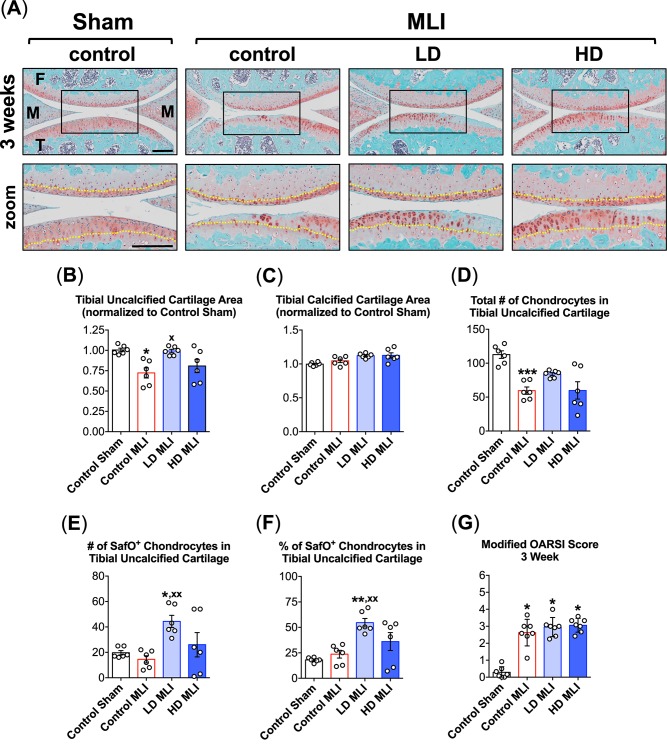
hCol1 is chondroprotective in the early stages of murine PTOA. Panel (A) presents an array of representative 40x Safranin O/Fast Green stained sagittal sections (40x) from the medial compartment of sham and MLI joints 3 weeks post-injury under various treatment conditions (control = vehicle, LD = 3.8mg hCol1/day, HD = 38mg hCol1/day). Joint structures are labeled (F = femur, M = meniscus, T = tibia) and the black box denotes the area shown in the zoomed images, where the tidemarks are denoted with a yellow dashed line. Black scale bars depict 100μm. Cartilage architecture was evaluated using the Osteomeasure System to determine the tibial uncalcified cartilage area (B), tibial calcified cartilage area (C), the number of chondrocytes in the tibial uncalcified cartilage (D), and the number (E) and percentage (F) of Safranin-O positive (SafO^+^) chondrocytes in the tibial uncalcified cartilage. OARSI scoring of the sections analyzed by histomorphometry was also performed (G). For histomorphometry and cell counting, symbols (○) represent the average measurement made from 3 sections/joint. For OARSI Scoring, symbols (○) represent the average score for each joint based on scoring of 3 sections/joint by four observers. Bars in all graphs represent the average for each experimental group (± SEM, N = 6). Significant differences between experimental groups in the histomorphometry data (B-F) were identified via one-way ANOVA with a Tukey’s multiple comparisons post-test (*p<0.05, **p<0.01, ***p<0.001 compared to Control Sham; ^**x**^p<0.05, ^**xx**^p<0.01 compared to Control MLI). Significant differences between experimental groups in the OARSI data (G) were identified via a Kruskal-Wallis Test with a Dunn’s multiple comparisons post-test (*p<0.05, compared to Control Sham).

While the automated histomorphometry approach was useful in identifying protective effects of hCol1 in PTOA, the underlying changes in cartilage architecture and cellularity that contributed to the overall preservation of total tibial cartilage were not discernable using this method. Thus, additional analyses were performed on Safranin O/Fast Green- or Alcian Blue Hematoxylin/Orange G-stained sections using a manual histomorphometry approach (Osteomeasure system) as well as standardized OARSI cartilage scoring. At 3 weeks post-MLI, there were observable differences in cartilage architecture between experimental groups, including apparent preservation of tibial uncalcified cartilage and cellularity in this zone in mice supplemented with hCol1. Consistent with the automated analysis (Fig 2B), reduction of tibial uncalcified cartilage area following MLI was mitigated significantly by hCol1 supplementation, with the LD group showing significant effects and the HD trending in that direction (Fig 3B). Regarding calcified cartilage area (tide mark to osteochondral junction), MLI had no effect and neither did hCol1 supplementation (Fig 3C). Loss of chondrocytes from the tibial uncalcified zone following MLI appeared to be reduced in LD and HD hCol1 fed animals (Fig 3A), but this trend failed to achieve statistical significance (Fig 3D). Suggesting that hCol1 supports chondrocyte proteoglycan matrix production at the 3 week time point post-MLI, the number and percentage of Safranin O-positive chondrocyte lacunae were significantly increased in LD hCol1-supplemented mice, with the HD group trending in that direction (Fig 3E and 3F). Finally, the impact of MLI on the joint cartilage 3 weeks post injury was significant based on OARSI scoring, but the generalized analysis of cartilage content using this scoring system was not sensitive enough to identify any improvement in the hCol1 supplemented groups (Fig 3G). It should be noted that there were no significant differences between experimental groups in other structural analyses performed, including uncalcified and calcified cartilage area on the femoral condyles and the number of hypertrophic chondrocytes, and in any zone of the cartilage on the femoral condyles and tibial plateaus (data not shown). Also of note, the apparent inability of HD hCol1 to match the efficacy of LD hCol1 in preserving uncalcified cartilage and Safranin O-positive chondrocyte populations at 3 weeks post-MLI may be due to two outlier samples in the HD group that caused a large error (Fig 2B, 2E & 2F).

**Fig 4:**
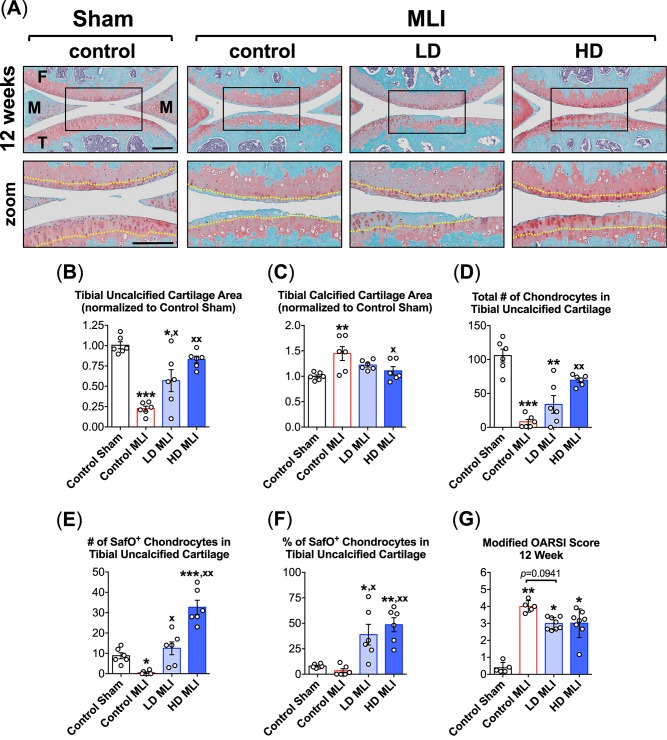
hCol1 protects against cartilage loss in mid to late stage murine PTOA. Panel (A) presents an array of representative 40x Safranin O/Fast Green stained sagittal sections from the medial compartment of sham and MLI joints 12 weeks post-injury under various treatment conditions (control = vehicle, LD = 3.8mg hCol1/day, HD = 38mg hCol1/day). Joint structures are labeled (F = femur, M = meniscus, T = tibia) and the tidemarks are denoted with a yellow dashed line in the zoomed images. Black scale bars depict 100μm. Cartilage architecture was evaluated using the Osteomeasure System to determine the tibial uncalcified cartilage area (B), the tibial calcified cartilage (C), the number of chondrocytes in the tibial uncalcified cartilage (D), and the number (E) and percentage (F) of Safranin-O positive (SafO^+^) chondrocytes in the tibial uncalcified cartilage. OARSI scoring of the sections analyzed by histomorphometry was also performed (G). For histomorphometry and cell counting, symbols (○) represent the average measurement made from 3 sections/joint. For OARSI Scoring, symbols (○) represent the average score for each joint based on scoring of 3 sections/joint by four observers. Bars in all graphs represent the average for each experimental group (± SEM, N = 6). Significant differences between experimental groups in the histomorphometry data (B-F) were identified via one-way ANOVA with a Tukey’s multiple comparisons post-test (*p<0.05, **p<0.01, ***p<0.001 compared to Control Sham; ^**x**^p<0.05, ^**xx**^p<0.01 compared to Control MLI, N = 6). Significant differences between experimental groups in the OARSI data (G) were identified via a Kruskal-Wallis Test with a Dunn’s multiple comparisons post-test (*p<0.05, **p<0.01 compared to Control Sham).

A similar set of manual histomorphometric analyses were performed in Safranin O/Fast Green-stained sagittal joint sections harvested 12 weeks post-MLI. At this time point, hCol1 appeared to provide more significant protection than at 3 weeks, with representative sections depicting apparent preservation of articular cartilage area and cellularity (Fig 4A). Use of Osteomeasure again facilitated quantification of key cartilage structural elements, with clear hCol1 dose-dependent preservation of uncalcified tibial cartilage following MLI (Fig 4B). Complimenting this effect was a protection against MLI-induced expansion of the tibial calcified cartilage zone, with joints from HD hCol1-treated mice showing a significant reduction in calcified cartilage area (Fig 4C). Regarding cellularity, MLI-induced nearly complete loss of chondrocytes in the uncalcified tibial cartilage by 12 weeks, with hCol1 dose dependently protecting against this effect (Fig 4D). Remarkably, chondrocyte number in the HD hCol1 group was not significantly different from sham operated control group, indicating substantial efficacy of the supplement in preserving cartilage cellularity. Regarding the number and percentage of Safranin O-positive chondrocyte lacunae in the tibial uncalcified cartilage, the nearly complete loss of cells producing proteoglycan matrix 12 weeks post-MLI was fully rescued in mice supplemented with hCol1 (Fig 4E and 4F). In fact, suggestive of a chondroregenerative effect, both LD and HD hCol1 groups displayed both a greater number and greater percentage of Safranin O-stained lacunae in tibia uncalcified cartilage compared to Control Sham joints (Fig 4E and 4F). While, as expected, the OARSI score was significantly increased 12 weeks post-MLI, hCol1 supplemented mice only showed modest improvement of the score, with the LD group trending toward significance (Fig 4G). As discussed for analytics performed on joints at the 3 week time point, it should be noted that there were no significant differences between experimental groups in uncalcified and calcified cartilage area on the femoral condyles, and in the number of hypertrophic chondrocytes in any zone of the cartilage on the femoral condyles and tibial plateaus (data not shown).

### PTOA-induced upregulation of MMP13 is ameliorated by hCol1

**Fig 5:**
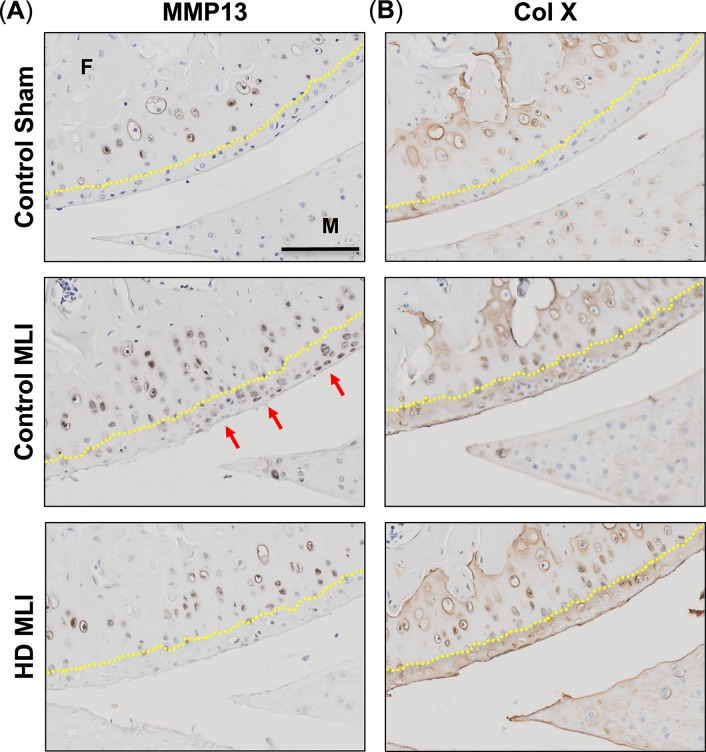
hCol1 reduces MMP13 levels in articular cartilage of mice following MLI. 3 weeks post-injury (Sham or MLI), knee joints were harvested from mice and hypertrophic chondrocytes were analyzed by immunohistochemistry of MMP13 and ColX. Representative sagittal sections depict (A) MMP13 and (B) ColX stained chondrocytes (brown) with cell nuclei counterstained with hematoxylin (blue). Yellow dasted lines highlight the tide mark, separating calcified cartilage from uncalcified cartilage. Joint structures are labeled (F = femur, M = meniscus), and the black scale bar depicts 100μm.

An important step in cartilage degeneration is the aberrant transition of articular chondrocytes into the hypertrophic state, an event marked by the expression of catabolic enzymes such as MMP13, and the marker of terminal hypertrophy, Type 10 Collagen (ColX). Accordingly, it is possible that the chondroprotective action of hCol1 documented in Figs 2–4 is associated with an inhibition of chondrocyte hypertrophy. To address this question, immunohistochemistry was performed to evaluate MMP13 and ColX protein levels in the articular cartilage. As expected, and indicative of chondrocyte hypertrophy, Control MLI mice had elevated levels of both MMP13 and ColX in the uncalcified cartilage compared to Control Sham mice at the 3 week time point (Fig 5A and 5B). Mice supplemented with HD hCol1 were protected from the MLI induced MMP13 staining in the uncalcified cartilage, suggesting that preservation of cartilage architecture in this group could in part be due to inhibion of matrix degeneration (Fig 5A). In contrast, ColX staining was not reduced in MLI mice supplemented with hCol1, indicating that hCol1 did not impact terminal chondrocyte hypertrophy (Fig 5B). Overall, no differences were observed in the calcified cartilage, as chondrocytes in this zone were all actively producing similar levels of both MMP13 and ColX (Fig 5A and 5B). It should be noted that at the 12 week time point, MLI joints from control-supplemented mice had lost most of the uncalcified cartilage and associated chondrocytes, making comparisons with the treated groups uninformative (data not shown). Together, these data suggest that mice supplemented with the HD hCol1 may have been protected from cartilage degeneration in part due to reduced MMP13 production by chondrocytes residing in the uncalcified cartilage.

### Impact of hCol1 supplements on articular chondrocyte life cycle

**Fig 6:**
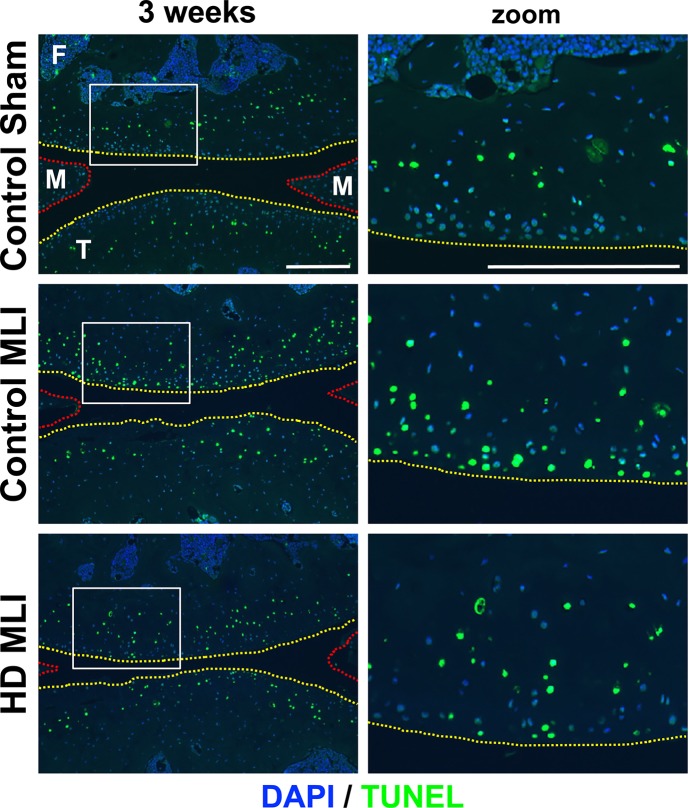
Chondrocyte apoptosis post-MLI is reduced in hCol1-supplemented mice. Joints were harvested from mice 3 weeks post-injury (Sham or MLI) and apoptotic cells were identified via TUNEL staining. Representative 100x sagittal sections (right column of panels) show the overall scope of cellular apoptosis (green), with all cell nuclei DAPI labeled (blue). The yellow dashed lines depict the articular cartilage surface and the red dashed lines outline the anterior and posterior horns of the meniscus. The region demarcated with the white box is magnified in the right column (zoom). White scale bars depict 100μm.

The remarkable ability of hCol1 supplementation to protect against chondrocyte loss in murine PTOA (Fig 4D) could be a key driver of its chondroprotective effect by setting the stage for persisting chondrocytes to produce matrix molecules (Fig 4E and 4F). To determine if the protection against MLI-induced chondrocyte loss was due to stimulation of proliferation or inhibition of apoptosis, representative tissue sections were analyzed via PCNA and TUNEL staining. While there was no detectable difference in the number of proliferative chondrocytes between experimental groups at 3 weeks post-MLI based on PCNA immunodetection (data not shown), MLI-induction of broad chondrocyte apoptosis was reduced 3 weeks post-MLI in mice supplemented with hCol1 (Fig 6). Representative TUNEL-stained sections showed a marked reduction in the number of apoptotic chondrocytes in superficial-to-middle layer cartilage zones (zoom panels in Fig 6). It should be noted that analysis was not informative at the 12 week time point because MLI joints in the control-supplemented mice were TUNEL-negative due to broad apoptotic loss of chondrocytes that had occurred prior to the harvest. Overall, these data suggest that the significantly larger number of chondrocytes seen in the uncalcified cartilage of hCol1 supplemented mice at 12 weeks post-MLI (Fig 4D) could be due to protection from apoptosis earlier in the disease process.

### Oral delivery of hCol1 protects against PTOA-induced synovial change

**Fig 7:**
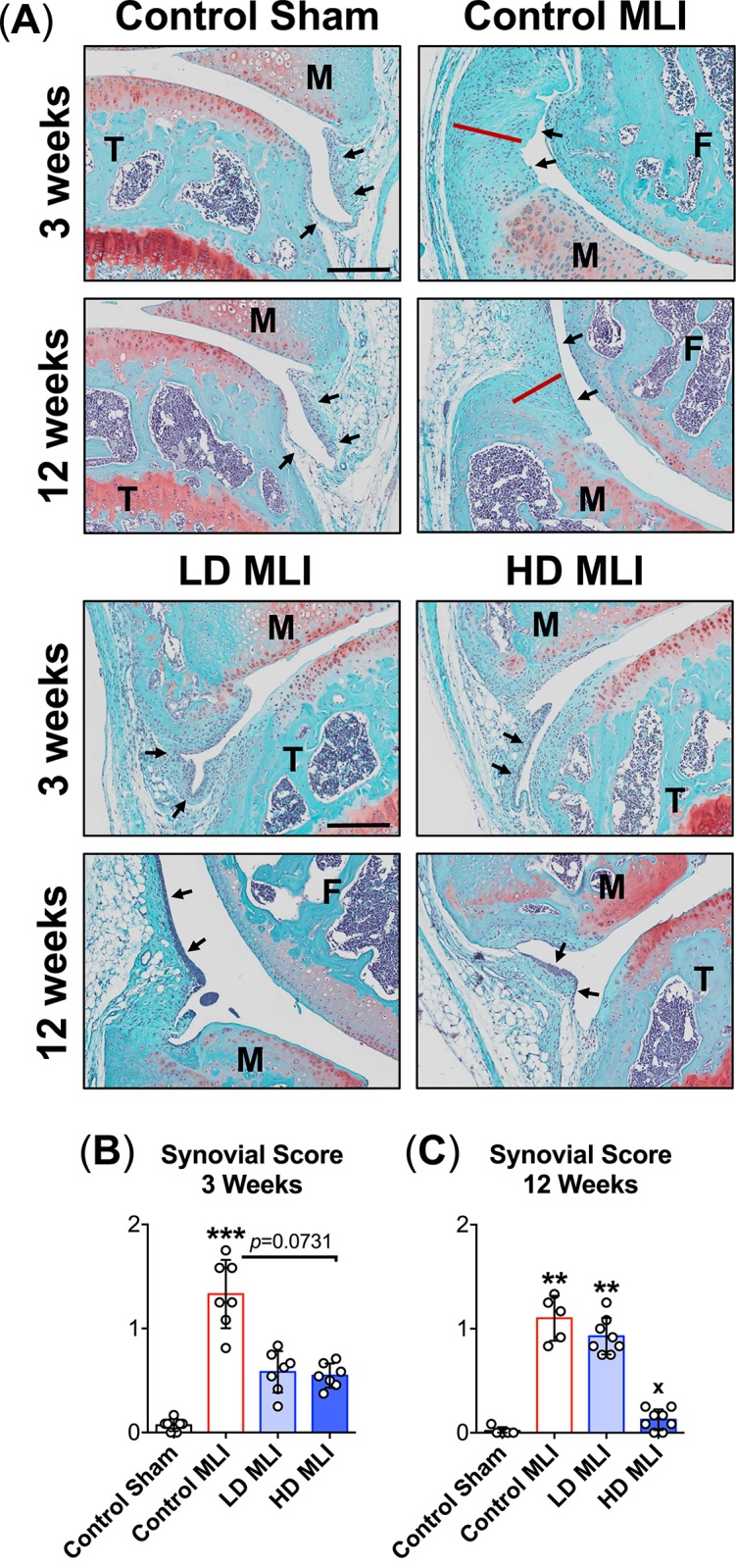
Synovial hyperplasia is reduced in mice supplemented with hCol1. (A) Tissue sections stained with Safranin O/Fast Green were used to examine the synovium. Representative 40x sagittal sections from Sham and MLI joints of mice supplemented with Control (vehicle, hazelnut cream), LD hCol1 or HD hCol1 that were harvested at 3 and 12 weeks post-injury are depicted. Joint structures are labeled (F = femur, M = meniscus, T = tibia), and synovial membranes are demarcated with black arrows. The red line highlights the thickness of hyperplastic synovium in the Control MLI section and the black scale bar depicts 100μm. A synovial scoring method was also employed to quantify synovial hyperplasia at both 3 weeks (B) and 12 weeks (C) post-injury. Symbols (○) represent the average Synovial Score for each joint based on scoring of 3 sections/joint by four observers. Bars represent the average Synovial Score per experimental group (± standard deviation, N = 5–8 joints). Significant differences between experimental groups were identified via a Kruskal-Wallis Test with a Dunn’s multiple comparisons post-test (*p<0.05, **p<0.01, ***p<0.001 compared to Control Sham).

The marked preservation of matrix-producing chondrocytes post-MLI in hCol1 supplemented mice begged for subsequent investigation of the impact of hCol1 on inflammatory synovial changes that are known to contribute to chondrocyte death following joint injury [32, 33]. Comparison of representative Safranin O/Fast Green stained sagittal knee joint sections revealed an apparent reduction in the severity of synovial hyperplasia in mice provided the hCol1 supplements. At both 3 and 12 weeks post-MLI, synovial thickness was increased compared to Control Sham joints, while mice provided hCol1 were protected from MLI-induced hyperplastic synovial change (Fig 7A). Synovial phenotypes were further investigated using a synovial scoring method, revealing significant effects in mice provided hCol1 supplements that were consistent with the representative histology. As expected, MLI induced a significant increase in synovial score (i.e. more robust synovial hyperplasia) at both 3 weeks (Fig 7B) and 12 weeks (Fig 7C) post-MLI. At 3 weeks, synovial scores trended toward a reduction (improvement) in the MLI groups that were supplemented with hCol1 (Fig 7B). Comparatively, at 12 weeks post-MLI, the synovium in mice from the HD hCol1 group was significantly less hyperplastic, suggesting that hCol1 supplements effectively reduced synovial/joint inflammation that is present in PTOA.

**Fig 8:**
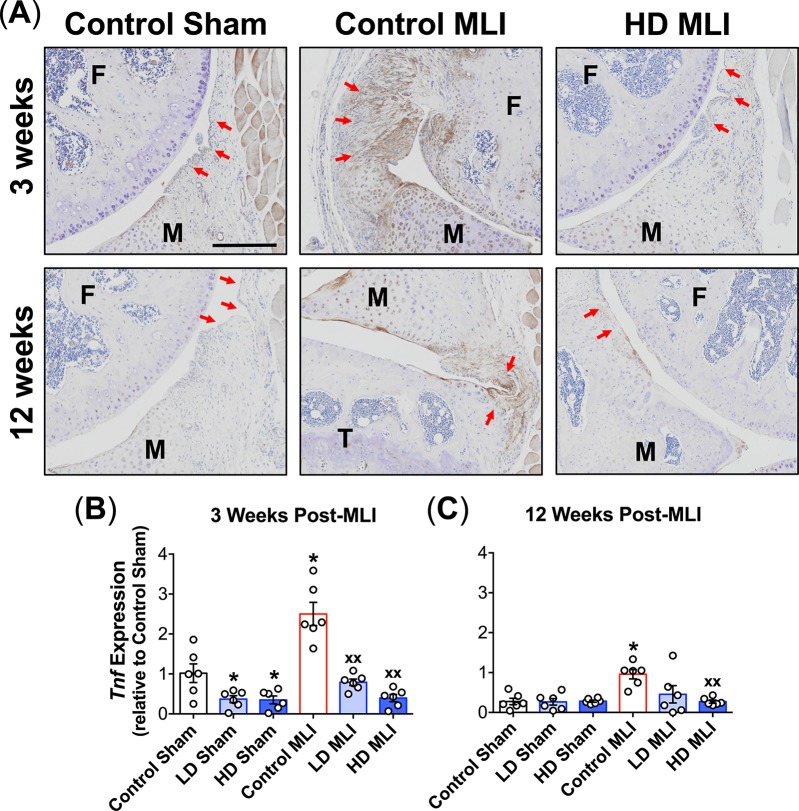
Post-injury upregulation of TNF in the synovium is reduced in mice supplemented with hCol1. (A) Representative TNF immunostained sagittal sections (100x) from Sham and MLI joints of mice supplemented with Control (vehicle, hazelnut cream), LD hCol1 or HD hCol1 that were harvested at 3 and 12 weeks post-injury are shown. Joint structures are labeled (F = femur, M = meniscus, T = tibia), synovial membranes are demarcated with red arrows, and brown staining of the tissue indicates intensity and location of TNF expression. The black scale bar depicts 100μm. mRNA was purified from synovial tissue collected from a separate cohort of similarly-treated mice at 3 weeks (B) and 12 weeks (C) post-injury. qRTPCR was performed to quantify *Tnf* expression level. Symbols (○) represent the *Tnf* level in each synovial sample and bars represent the average *Tnf* level for each experimental group (± SEM, N = 6). Significant differences between groups were identified via one-way ANOVA with a Tukey’s multiple comparisons post-test (*p<0.05 compared to Control Sham, ^xx^p<0.01 compared to Control MLI).

Among several cytokines implicated in diarthrodial joint degeneration, TNF is established as a central inflammatory mediator that is present in synovial fluid and upregulated in hyperplastic synovium in PTOA, particularly in the early post-trauma time frame [34, 35]. To investigate the impact of hCol1 supplementation on TNF expression post-MLI, both immunohistochemical staining and mRNA analysis of synovial membranes were performed. Representative immunostained sagittal sections revealed increased TNF expression at both 3 and 12 weeks post-MLI, with mice from the HD hCol1 cohort substantially protected from this effect (Fig 8A). This was confirmed via quantitative analysis of *Tnf* mRNA levels in synovial membranes harvested from the various experimental groups. Specifically, 3 weeks post-injury, both LD and HD hCol1 supplementation significantly reduced *Tnf* levels in synovium from both the sham and MLI cohorts (Fig 8B). While the overall synovial level of *Tnf* was lower 12 weeks post-injury, hCol1 still effectively reduced synovial *Tnf* in MLI joints, with the HD group achieving statistical significance (Fig 8C). It should be noted that while the synovial expression of other genes, including *Il1β*, *Prg4* and *Mmp13*, was increased following injury, particularly at 12 weeks post-MLI, hCol1 supplementation did not have a significant impact on expression level (data not shown). Overall, these findings suggest that dietary supplementation with hCol1 reduces synovial hyperplasia and mitigates synovial *Tnf* expression in both early and mid-stage PTOA.

## Discussion

In this study, experiments were carried out to investigate the impact of orally delivered hCol1 on the joint degenerative process in a mouse model of PTOA. Close examination of cartilage architecture, chondrocyte populations and synovial change revealed significant chondroprotective and anti-inflammatory effects in injured joints at early and mid-stages of OA degeneration. These novel findings support the hypothesis that daily oral consumption of hCol1 is joint protective and disease modifying in OA joints.

The concept that oral consumption of matrix components from cartilage and connective tissue may be effective in treating arthritic conditions has been debated since the mid 1980s. Among these agents, the most widely recognized, heavily studied, and broadly available are glucosamine and chondroitin sulfate, with published evidence suggesting that they are joint protective in OA based on work in animal models [36] and humans [37–40]. Fueling the efficacy debate, the widely cited Glucosamine/Chondroitin Sulfate Arthritis Intervention Trial known as GAIT concluded that these agents are not symptom-relieving or disease modifying in human OA [41]. Despite these and other conflicting reports, and without definitive preclinical study of joint tissue effects, glucosamine and chondroitin sulfate have been continued to be widely marketed as nutraceuticals that are joint protective and symptom relieving in various types of arthritis [42–44]. Study and use of these molecules has also opened the door to the development of other molecules of similar origin that likewise are thought to impact joint health, including collagens in their native state or as hydrolysates.

In the case of collagen-based nutraceuticals, numerous formulations have been developed which are marketed as dietary supplements or as additives or ingredients in the food industry. They are gaining popularity due to their documented positive effects on skin and bone. Specifically, collagen peptides enhance skin hydration and elasticity and stimulate the production of dermal matrix [17, 45]. In the skeleton, collagen peptides have been shown to increase bone strength and mineral density, supporting their potential disease modifying capability in osteoporosis [16, 46, 47]. Since collagen is a major component of the matrix in key joint structures including the articular cartilage, collagen peptides have also been proposed as potential disease modifiers in arthritic conditions [20]. Early work sparking this idea reported that oral administration of native type 2 collagen was effective at mitigating arthritic symptoms in animal models of rheumatoid arthritis (RA) [48, 49], with a human clinical trial demonstrating analogous protection in RA patients orally consuming chicken type 2 collagen [50]. A series of reports further substantiate this idea, demonstrating that various preparations of orally administered type 2 collagen effectively ameliorate collagen-induced arthritis in mice [51], in obese arthritic dogs [36], and in equine OA [52]. Remarkably, they have also been shown to have therapeutic efficacy in managing symptoms in human OA [53]. Similarly, oral supplementation with a type 1 collagen hydrolysate has been shown to structurally improve the thickness of articular cartilage in OA via gadolinium-enhanced MRI [54], suggesting potential disease-modifying capability of this agent. hCol1, a specific collagen-based product marketed by Rousselot under the trade name Peptan^®^, has also recently been shown by Jiang et al. to reduce joint pain in a prospective, randomized, double blind, placebo-controlled clinical trial in elderly women with mild to moderate knee OA [21]. This study, which reported improved Western Ontario and McMaster Universities Arthritis Index (WOMAC) and Lysholm scores in subjects consuming 8gm Peptan per day for 6 months, establishes the idea that hCol1 supports joint health and reduces joint pain during early to mid-stage OA. Given that little is known about the biological action of hCol1 at the tissue and cellular level, we executed experiments to elucidate its impact on joint structure in a preclinical model of PTOA.

An experimental protocol was carried out in this study that involved daily oral delivery of hCol1 to mice that were induced to develop PTOA via surgical administration of a meniscal-ligamentous injury (Fig 1). Two doses of hCol1 were chosen, with the higher dose (38mg/day) set to be the body weight equivalent to the human dose (7.4g/day) that was employed in the Jiang et al. clinical study mentioned above [21]. To investigate the hypothesized joint-protective impact of long term use, mice were supplemented with hCol1 for a one month pre-treatment period, followed by administration of MLI surgery and subsequent assessment of joints. Structural and cellular evaluation of the articular cartilage of sham and injured knee joints using both automated and manual histomorphometric methods revealed that while there were no discernable cartilage effects in the sham/normal joints, at early and later time points in the development of OA, mice administered hCol1 were chondroprotected, particularly at 12 weeks post-injury. Uncalcified tibial articular cartilage was preserved and the articular chondrocyte population was maintained (Figs 2, 3 and 4), possibly via reduction of MMP13 and inhibition of chondrocyte apoptosis (Figs 5 and 6, respectively). Regarding apoptosis specifically, its inhibition was particularly apparent on the femoral condyles (Fig 6), which correlated with the complete protection from cartilage loss in this region of the joint (Fig 4). Interestingly, chondrocyte populations were not only preserved, but the number of cells actively producing Safranin O stained proteoglycan matrix was increased significantly in the hCol1-treated groups at both time points. These results parallel published work suggesting chondrogenic and cartilage matrix anabolic effects of hCol1 in adipose tissue derived stem cells [19], and the anabolic effects of type 1 collagen hydrolysate and prolyl-hydroxyproline [55, 56] and collagen peptide fragments [57] on chondrocyte matrix production.

In addition to the remarkable cartilage and chondrocyte effects observed in the degenerating knee of mice supplemented with hCol1, significant changes were also observed in the synovium. While there were no discernable synovial effects of the supplements in sham operated joints, synovial hyperplasia was evident at both early and late time points following injury (3 and 12 weeks respectively), with semi-quantitative scoring revealing significant protection from this hyperplasia in mice supplemented with hCol1 (Fig 7). This protection was accompanied by reduced TNF protein and mRNA expression, suggesting an anti-inflammatory effect of hCol1 on the OA joint (Fig 8). This protection seemed to be restricted to TNF; synovial mRNA expression of other cytokines and catabolic factors known to participate in articular cartilage degeneration, including *Il1b*, *Mmp13* and *ADAMTS5*, were not affected in the hCol1 supplemented groups (data not shown). While hCol1 and other similar collagen-based supplements have not been shown to protect against the production of inflammatory mediators in joint tissue, there is evidence of anti-inflammatory effects of type 1 collagen hydrolysate and peptides in cardiovascular disease [58] and in ulcerative colitis [59]. Of note, the anti-inflammatory effects of oral hCol1 in the synovium described in this report parallel the anti-inflammatory effects that have been reported for glucosamine, chondroitin sulfate and glycosaminoglycans (reviewed in [44, 60]). Overall, when considered in conjunction with the observed cartilage structure modifications, daily oral supplementation with hCol1 has significant potential as a cartilage protective and anti-inflammatory agent in the treatment of OA.

The detailed analysis of joint structure that was completed in this study represents a novel attempt to understand the influence of a dietary supplement on OA joint degeneration specifically. The numerous soft tissue matrix-based oral supplements and nutraceuticals described above, including collagen-based agents, glucosamine, glycosaminoglycan, and chondroitin/keratin sulfate, have been studied in vitro (chondrocyte and synovial cultures) and/or in animal and human OA with pain assessment representing a primary measure of efficacy. Missing from many of these studies is the direct examination of the impact of these agents on cartilage architecture, chondrocyte populations and synovial status in animal models of disease. To definitively be classified as disease modifying, these structural analyses must be performed to establish tissue-level impact; it is for this reason that a detailed study of joint structure was pursued in this investigation. The positive effects of hCol1 on articular cartilage architecture and synovial hyperplasia that are presented in this report not only provide a structural basis for the mitigation of pain that has been reported in OA patients [21], but they also provide a strong rationale to further evaluate hCol1 effects on joint structure in the context of human disease.

An open question that is yet to be answered for hCol1 and other nutraceuticals that are purported to be joint protective, including type 2 collagen-based preparations, glucosamine and chondroitin sulfate, relates to their mechanism of action. The dominant hypothesis posits that these agents, or their digested fragments, are absorbed across the intestinal lumen, enter the blood stream and have direct effects on articular chondrocytes or other cells in the joint. Supporting this general idea, regarding collagen peptides specifically, hydroxyproline-containing di- and tri-peptides are detectable in the human serum after oral consumption [13, 61], with 90% of consumed peptides absorbed within 6 hours in mice [62]. Consistent with this, we detected a significant dose dependent surge in serum hydroxyproline levels in mice that were supplemented with hCol1 (Fig 1). Serum hydroxyproline was highest when blood was sampled within 1 hour of consumption (Fig 1E), although the surge was still detectable at 3 hours in the high dose hCol1 group (Fig 1D). Once in the serum, these peptide fragments presumably have access to all organ systems, with one report confirming distribution of a radiolabeled preparation to skeletal elements, muscle, skin and cartilage in rats [63]. Another study performed in rats provides autoradiographic evidence for the presence of radiolabeled proline-hydroxyproline dipeptide in articular cartilage and synovium within 30 minutes of ingestion [64]. As mentioned earlier in this report, there are also in vitro data indicating that various types of collagen peptides can directly affect chondrocyte function [19, 45, 55, 57], supporting the hypothesis that absorbed collagen peptide fragments could directly influence cell behavior in the joint. While this line of reasoning lays out a basis for orally ingested collagen peptides or cartilage matrix molecules to impact joint tissues, the nature of the peptides/compounds that actually make it to the joint is not known, and the pathway of action, whether it be in the chondrocyte, synoviocyte, or other cell in the joint, can only be speculated. Current effort in the field aims at addressing these questions.

## Conclusions

The efficacy of nutraceuticals comprised of cartilage matrix components in supporting joint health and protecting against OA progression has been tested and debated over the past two decades. Dietary supplements containing chondroitin and glucosamine are marketed worldwide and have been evaluated in human OA clinical trials, with mixed results. With both in vitro and anecdotal animal and human data suggesting that oral consumption of hCol1 could represent a strategy for supporting joint health, a preclinical study was executed to definitively determine the impact of this agent on the cartilage degenerative process that plays out in a murine model of PTOA. Results reported here suggest that daily consumption of hCol1 protects against cartilage loss and stimulates the production of proteoglycan by chondrocytes in injured joints. Articular chondrocyte number is also increased in the joints of mice from the hCol1 groups, possibly due to an inhibition of apoptosis in these cells. Trauma-related synovial hyperplasia is also reduced in supplemented mice, and this effect occurs in conjunction with reduced synovial TNF expression. Overall, these results suggest that hCol1 is chondroprotective and anti-inflammatory in posttraumatic OA, setting the stage for further mechanistic study and evaluation of joint structural modifications and potential disease modifying effects in a human clinical trial.
